# Serum D-dimer should not be used in the diagnosis of venous thromboembolism within 28 days of total knee replacement surgery

**DOI:** 10.1186/s43019-020-00068-x

**Published:** 2020-09-22

**Authors:** Ethan Toner, Tobenna Oputa, Heather Robinson, Olivia McCabe-Robinson, Andrew Sloan

**Affiliations:** 1 Trauma & Orthopaedic Department, Royal Victoria Hospital, 274 Grosvenor Rd, Belfast, BT12 6BA UK; 2 Trauma & Orthopaedic Department, Royal Blackburn Hospital, Blackburn, UK

**Keywords:** Total knee replacement, D-dimer, Venous Thromboembolism, Deep vein thrombosis (DVT)

## Abstract

**Background:**

Serum D-dimer is frequently used to rule out a diagnosis of venous thromboembolism (VTE), a recognised complication following total knee replacement (TKR). TKR is known to cause a rise in D-dimer levels, reducing its specificity. Previous studies have demonstrated that D-dimer remains elevated within 10 days of TKR and therefore should be avoided. The aim of this study was to determine whether serum D-dimer tests are clinically appropriate in identifying VTE when performed within 28 days of TKR.

**Methods:**

Case notes for patients who had a serum D-dimer test performed for clinically suspected VTE at ≥ 28 days following TKR were retrospectively reviewed for a 6-year period. Demographics, D-dimer result, time after surgery and further radiological investigations were recorded.

**Results:**

Fifty patients underwent D-dimer tests at ≥ 28 days following surgery (median 60 days, range 29–266); 48 of these patients had a positive result. Of these, five had confirmed VTE on radiological investigations. Serum D-dimer was raised in 96% of the patients. Only 10.42% of these patients had confirmed VTE. No patients with negative D-dimers had confirmed VTE.

**Conclusions:**

These findings suggest that serum D-dimer remains raised for at least 28 days and possibly considerably longer following TKR. Serum D-dimer should not be used in patients with clinically suspected VTE within this period because of its unacceptably low specificity of 4.44% and positive predictive value of 10.42%, which can lead to a delay in necessary further radiological investigations, waste of resources and unnecessary exposure to harm.

## Background

Venous thromboembolism (VTE) is a recognised complication of total knee replacement surgery (TKR). The incidence of post-operative VTE has been reported to be as high 40–60% in major orthopaedic surgery without anticoagulation [1] and as high as 10% within 3 months of surgery despite routine VTE prophylaxis [2]. Clinical diagnosis of a deep vein thrombosis (DVT) in the post-operative period can be challenging due to the expected swelling and pain following TKR, whilst other patients may remain asymptomatic [3]. Incorrectly diagnosed DVT or untreated DVT can bring about a life-threatening pulmonary embolus (PE) [3].

The use of serum D-dimer has been shown to assist in the diagnosis of DVT [1] due to its high sensitivity (> 95%) in selective patient groups [4, 5]. A negative result can be used to exclude a diagnosis of VTE. However, with a low specificity, which ranges from 30.5–36.5% [5], a diagnosis cannot be confirmed with a positive result [3, 6, 7], and further tests are frequently performed. Effective management relies on the early detection of DVT. In practice, ultrasonography is utilised for the diagnosis of DVT in the lower limb [6], whereas a computed tomography pulmonary angiogram (CTPA) or a ventilation-perfusion (VQ) scan can confirm the diagnosis of a PE [8].

**Fig. 1:**
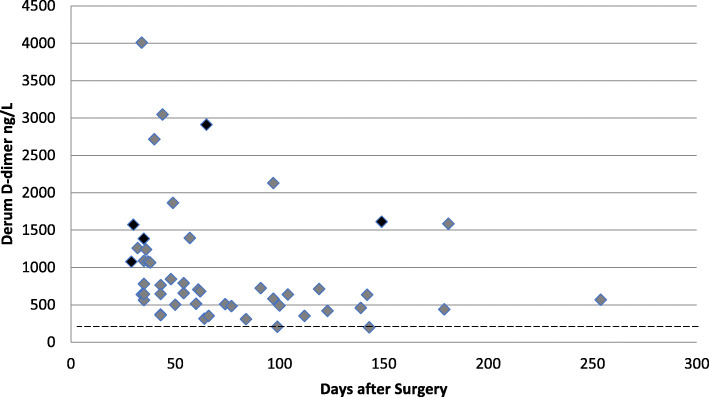
Serum D-dimer result plotted against time after surgery. Cases highlighted in black had confirmed VTE on radiological investigation. The dotted line shows the level where D-dimer result was considered positive

TKR is also known to cause a rise in serum D-dimer levels. Studies have demonstrated that after TKR, serum D-dimer levels remain elevated from day 0 to day 10 post-operatively [9, 10], with no significant difference in D-dimer level between patients with or without a DVT from day 0 to day 10 following surgery [3, 9, 10]. Bytniewski et al*.* [10] found that higher levels of serum D-dimers in the post-operative period of up to 10 days were not related to increased risk of VTE. Due to its low specificity in this period, the use of serum D-dimer within the first 10 days following TKR is not recommended as a screening tool for VTE [9]. However, no studies have examined the specificity of D-dimers after this period. The aim of this study was to calculate the specificity of serum D-dimer levels at ≥ 28 days after TKR to determine whether or not there is a role for serum D-dimer within the 28 days following surgery (Fig. 1).

## Method

Retrospective case note reviews were performed for a 6-year period from 2012 to 2018 across two district general hospitals in the United Kingdom to identify all patients who had a serum D-dimer performed at 28 days or longer following TKR for a suspected VTE. Twenty-eight days post-operatively was chosen as a cut-off date, which is a date used in previous studies reviewing the incidence of post-operative VTE in orthopaedic surgery. Post-operative DVT has previously been defined as one occurring within 4 weeks of surgery since the relationship between surgery and DVT is not clear beyond this time [11]. Patient’s underwent D-dimer testing if a clinical suspicion arose of a VTE because of signs of increased leg swelling, calf tenderness and or skin changes. A serum D-dimer > 245 ng/L was considered to be elevated and was therefore deemed a positive result [12, 13]. Patients with a positive D-dimer result who did not go on to have any further investigations were excluded. Demographic details, date of surgery, date of serum D-dimer test, result of serum D-dimer and result of any further radiological imaging were recorded. Diagnosis of VTE was based on radiographic evidence with ultrasonography or CTPA. When discrepancies were found, further analysis was carried out to look into the underlying key causes. All cases were searched for by three authors independently.

## Results

Seventy-one patients were identified as having serum D-dimer performed at ≥ 28 days following TKR for suspected VTE. Twenty patients were excluded from the study as per the exclusion criteria, either because they were lost to follow-up, because inadequate data were available or because treatment was based on clinical diagnosis alone without the use of further diagnostic imaging. One further patient was excluded because the serum D-dimer test was performed at 384 days after surgery, and the previous TKR was not believed to be a relevant factor when the clinical suspicion leading to the investigation was raised.

Of the remaining 50 eligible patients, 15 patients were male, and 35 were female. All patients had primary total knee replacements. The median age at the time of surgery was 70.41 years (range 53.34–86.01 years). Serum D-dimer was performed between 29 days and 266 days (median 60 days). Three patients were taking anticoagulants prior to surgery, 13 patients had a previous knee replacement, and five patients had a previous hip replacement. Forty-nine patients were prescribed anticoagulants post-operatively (47 low molecular weight heparin, 1 warfarin, and 1 aspirin) Mean length of stay was 5.26 days (range 1–20 days). Table 1 shows patient demographics and characteristics.

**Table 1:** Patient demographics and characteristics

Total number n.	50
Male n. (%)	15 (30%)
Female n. (%)	35 (70%)
Median Age at time of surgery (Range)	70.41 (53.34 – 86.10)
Previous knee Replacement n. (%)	13 (26%)
Previous hip Replacement n. (%)	5 (10%)
Patients taking anticoagulants prior to surgery n. (%)	4 (8%)
Post operative anticoagulation n (%)	
LMWH	47 (94%)
Aspirin	1 (2%)
Warfarin	1(2%)
None	1(2%)
Mean Length of Hospital Stay (Range)	5.26 days (1 – 20 days)
Comorbidities n. (%)	
Hypertension	15 (30%)
Asthma	10 (20%)
Hypothyroidism	9 (18%)
CKD	7 (14%)
Previous neoplasm	7 (14%)
Previous VTE	4 (8%)
Epilepsy	3 (6%)
Diabetes	3 (6%)
Angina /Ischaemic heart disease	3 (6%)
CVD	2 (4%)
Previous myocardial infarction	2 (4%)
Cerebrovascular disease	2 (4%)
Valvular Heart Disease	2 (4%)
Atrial Fibrillation	1 (2%)

Of the 50 patients, 48 patients (96%) had a positive serum D-dimer result, and only two patients had a negative serum D-dimer result. All patients, including those with a negative serum D-dimer, went on to have radiological investigations. Neither of the two patients with a negative serum D-dimer had confirmed VTE on radiological investigations. Of the 48 patients with a positive serum D-dimer, five (10.42%) had confirmed VTE on radiological investigations. One patient had an above-knee DVT, two patients had a below knee DVT, and two patients had a pulmonary embolus. Specificity was 4.44%, and the positive predictive value was 10.42%.

## Discussion

Serum D-dimer represents products of cross-linked fibrin degradation from fibrinolysis. Activation of the clotting cascade begins when bleeding occurs, leading to thrombus formation from fibrin, which halts the bleeding. Plasmin works to breakdown the thrombus, and consequently, the fibrin degradation products are released, giving rise to D-dimers. Raised levels of serum D-dimer represent increased fibrinolysis and active coagulation within the vasculature. However, increased levels of fibrin production and thus raised serum D-dimer levels are not exclusive for VTE. Raised D-dimer levels can be manifested in other conditions such as trauma, infection, surgery, cancer, inflammation, ischaemic heart disease, pregnancy and in elderly patients, to name a few [14–16].

Patients undergoing major surgery such as TKR can have a raised serum D-dimer secondary to activation of the fibrinolytic process and hence can lead to false positives for D-dimer tests in patients postoperatively, thus reducing the specificity of this test [3]. Tourniquet application is frequently used during TKR surgery, which can lead to reported complications such as increased VTE risk, skin blistering, muscle injury or the effects on post-operative functional recovery. Tourniquet use has been found to increase fibrinolysis and therefore increase D-dimer levels, although Aglietti et al. found this did not increase VTE risk [17]. Much dispute remains in the literature with many studies detailing various findings. Zhang et al. suggested that tourniquet use impacted on VTE risk for patients [18] whilst Fukuda et al. reported that the use of tourniquet was beneficial due to reduced blood loss and clearer surgical fields, and caused no increased risk of DVT when compared to TKR without a tourniquet [19].

Our results suggest that in patients with clinically suspected DVT within 28 days following TKR, the serum D-dimer has a specificity of 4.44% and a positive predictive value of 10.42%. These parameters make the use of serum D-dimer an unacceptable screening test in the diagnosis of VTE in patients within 28 days of TKR. With 96% of patient’s having a positive D-dimer and only 4% (at 99 days and 143 days post operatively) having a negative D-dimer, it would seem that in the vast majority of cases, the test simply leads to an unnecessary delay in further radiological investigations.

Many radiology departments require patients to have undergone screening for VTE with serum D-dimer testing prior to proceeding to radiological investigation. In a survey of UK hospitals, Rafe et al. showed that 51 radiology departments out of 70 in the UK required all patients undergoing radiological investigations for suspected VTE following TKR and total hip replacement to initially have serum D-dimer testing [3]. The laboratory testing of serum D-dimer in the initial post-operative phase leads to unnecessary venepuncture, which in turn can lead to patient stress, haematomas, bruising and phlebitis [20, 21]. Reducing unnecessary blood tests can also reduce costs and prevent wasting resources [22].

Previous studies to date have demonstrated that serum D-dimer testing up to 10 days after a total knee replacement should not be used as a diagnostic test [9, 10]. Our study suggests that D-dimer testing is a poor choice of investigation for VTE for up to 28 days after surgery and should be avoided and replaced with more accurate radiological investigations. Furthermore, the first negative D-dimer result was recorded at 99 days after surgery, suggesting that VTE remains raised for significantly longer than 28 days although we are not able to reliably draw such a conclusion from our data.

### Limitations

Our study has some limitations that must be recognised. Our study did not look at use of tourniquets, intra-articular injection of tranexamic acid or body mass index (BMI). These factors may influence serum D-dimer levels and the activation of clotting [17]. Total knee replacements are often carried out with the use of a tourniquet to provide a dry field and increase the ease of fixation for the surgeon. Tourniquet application has been shown to increase fibrinolysis and subsequently D-dimer levels [17]. Tranexamic acid can reduce blood loss and post-operative swelling from TKR surgery [23]. Raised BMI has also been shown to have an association with increased risk of VTE [24].

## Conclusion

Serum D-dimer has previously been demonstrated to be elevated from day 1 following TKR and remains raised for 10 days [9]. The results of our study suggest that serum D-dimer remains raised for at least 28 days and possibly considerably longer. Therefore, serum D-dimer should not be used in patients with clinically suspected VTE within this period, due to an unacceptably low specificity of 4.44% and a positive predictive value of 10.42%. Serum D-dimer is frequently used within this time frame. With 96% of patient’s having a positive D-dimer at 28 days or beyond, in most cases, this test leads to a delay in necessary further radiological investigations, is a waste of resources and unnecessarily exposes the patients to harm. Our results also suggest that the sensitivity may remain low for a significantly longer period than 28 days, and therefore, further research is needed to determine at what point serum D-dimer becomes an acceptable test following TKR. We therefore advocate that clinicians should not use the D-dimer test within 28 days of TKR and should also have caution about using this test after this period, instead proceeding to radiological investigation as first line when trying to rule out a diagnosis of post-operative VTE following TKR.

## Data Availability

Available on request.
